# Economic evaluation of the new oral anticoagulants for the prevention of thromboembolic events: a cost-minimization analysis: Avaliação econômica dos novos anticoagulantes para a prevenção de eventos tromboembólicos: análise de custo-minimização

**DOI:** 10.1590/1516-3180.2016.0019260216

**Published:** 2016-07-18

**Authors:** Milena Soriano Marcolino, Carisi Anne Polanczyk, Ana Carolina Caixeta Bovendorp, Naiara Silveira Marques, Lilian Azevedo da Silva, Cintia Proveti Barbosa Turquia, Antonio Luiz Ribeiro

**Affiliations:** I MD, MSc, PhD. Adjunct Professor, Department of Internal Medicine, Medical School, Universidade Federal de Minas Gerais (UFMG), Belo Horizonte, MG; Institutos Nacionais de Ciência e Tecnologia (INCT) para Avaliação de Tecnologia em Saúde (IATS), Brasília, DF, Brazil.; II MD, MSc, PhD. Adjunct Professor, Department of Internal Medicine, Medical School, Universidade Federal Rio Grande do Sul (UFRGS), Porto Alegre, RS; Institutos Nacionais de Ciência e Tecnologia (INCT) para Avaliação de Tecnologia em Saúde (IATS), Brasília, DF, Brazil.; III MD. Cardiology Resident, Instituto Dante Pazzanese de Cardiologia (IDPC), São Paulo, SP, Brazil.; IV MD. Attending Physician, Family Health Program, Belo Horizonte, MG, Brazil.; V Manager, Dispensing Pharmacy, Hospital Júlia Kubitschek (HJK), Belo Horizonte, MG, Brazil.; VI Nurse, Unimed-BH, Belo Horizonte, MG, Brazil.; VII MD, MSc, PhD. Full Professor, Department of Internal Medicine, Medical School, Universidade Federal de Minas Gerais (UFMG), Belo Horizonte, MG; Institutos Nacionais de Ciência e Tecnologia (INCT) para Avaliação de Tecnologia em Saúde (IATS), Brasília, DF, Brazil.

**Keywords:** Anticoagulants, Warfarin, Atrial fibrillation, Costs and cost analysis, Public health, Anticoagulantes, Varfarina, Fibrilação atrial, Custos e análise de custo, Saúde pública

## Abstract

**CONTEXT AND OBJECTIVE:**

Randomized clinical trials have shown that the new oral anticoagulants have at least similar impact regarding reduction of thromboembolic events, compared with warfarin, with similar or improved safety profiles. There is little data on real costs within clinical practice. Our aim here was to perform economic analysis on these strategies from the perspective of Brazilian society and the public healthcare system.

**DESIGN AND SETTING:**

Cost-minimization analysis; anticoagulation clinic of Hospital Municipal Odilon Behrens, Belo Horizonte, MG, Brazil.

**METHODS:**

Patients at the anticoagulation clinic were recruited between August and October 2011, with minimum follow-up of four weeks. Operational and non-operational costs were calculated and corrected to 2015.

**RESULTS:**

This study included 633 patients (59% women) of median age 62 years (interquartile range ­49-73). The mean length of follow-up was 64 ± 28 days. The average cost per patient per month was $ 54.26 (US dollars). Direct costs accounted for 32.5% of the total cost. Of these, 69.5% were related to healthcare professionals. With regards to indirect costs, 52.4% were related to absence from work and 47.6% to transportation. Apixaban, dabigatran and rivaroxaban were being sold to Brazilian public institutions, on average, for $ 49.87, $ 51.40 and $ 52.16 per patient per month, respectively, which was lower than the costs relating to warfarin treatment.

**CONCLUSION:**

In the Brazilian context, from the perspective of society and the public healthcare system, the cumulative costs per patient using warfarin with follow-up in anticoagulation clinics is currently higher than the strategy of prescribing the new oral anticoagulants.

## RESUMO

**CONTEXTO E OBJETIVO:**

Estudos clínicos randomizados demonstraram que novos anticoagulantes orais têm pelo menos impacto semelhante em reduzir eventos tromboembólicos quando comparados à varfarina, com perfil de segurança similar ou superior. Há pouca evidência acerca de custos reais na prática clínica. Nosso objetivo é realizar análise econômica dessas estratégias, na perspectiva do sistema de saúde pública e da sociedade brasileiros.

**TIPO DE ESTUDO E LOCAL:**

Análise de custo-minimização; Clínica de Anticoagulação do Hospital Municipal Odilon Behrens, Belo Horizonte, MG, Brasil.

**MÉTODOS:**

Os pacientes da clínica de anticoagulação foram recrutados de agosto a outubro de 2011, com tempo mínimo de acompanhamento de quatro semanas. Custos operacionais e não operacionais foram computados e corrigidos para 2015.

**RESULTADOS:**

Este estudo incluiu 633 pacientes, com idade mediana de 62 (intervalo interquartil 49-73) anos, sendo 59% mulheres. O tempo médio de acompanhamento foi de 64 ± 28 dias. O custo médio por paciente por mês foi de $ 54.26 (dólares). Custos diretos foram responsáveis por 32,5% do custo total. Destes, 69,5% foram relacionados aos profissionais de saúde. Em relação aos custos indiretos, 52,4% estavam relacionados ao absenteísmo ao trabalho e 47,6% ao transporte. Apixaban, dabigatran e rivaroxaban são vendidos a órgãos públicos brasileiros, respectivamente, a um preço médio mensal de $ 49.87, $ 51.40 e $ 52.26 por paciente por mês, valores inferiores aos custos relacionados ao tratamento com varfarina.

**CONCLUSÃO:**

No contexto brasileiro, na perspectiva do sistema de saúde pública e da sociedade, os custos cumulativos por paciente em uso de varfarina acompanhados em clínica de anticoagulação são atualmente superiores à estratégia de prescrever novos anticoagulantes orais.

## INTRODUCTION

Atrial fibrillation is the most common sustained arrhythmia in clinical practice. It is associated with increased risk of stroke, systemic embolism, heart failure and mortality.^1^^,^^2^ Occurrences of stroke relating to atrial fibrillation are usually more severe, with a more extensive affected area, greater mortality and poorer functional outcome, in comparison with patients without atrial fibrillation.^3^ The current treatment for atrial fibrillation focuses on estimating the risk of cardioembolic events, in order to assess the need for anticoagulation, rate control, rhythm control in some symptomatic individuals and aggressive modification of cardiovascular risk.^1^ Use of anticoagulant therapy is effective in reducing the incidence of stroke, systemic embolism and mortality.^4^

A recent study that enrolled approximately 300,000 Brazilian primary care patients showed a prevalence of atrial fibrillation similar to that observed in developed countries, with a very low proportion of patients taking anticoagulants.^5^ The possible explanations for this underutilization are the lack of doctors in primary care with experience of managing patients with atrial fibrillation and making risk assessments on cardioembolic events; fear of the risk of bleeding complications such as intracranial hemorrhage; and limitations associated with the use of vitamin K inhibitors, such as the need for frequent dose control and adjustment in accordance with the prothrombin time and the international normalized ratio (INR), as well as the interactions of these inhibitors with drugs and food. Anticoagulation clinics are specialized clinics with multidisciplinary composition, which have a dual mission: to ensure patient education and information in accordance with a structured program that is adapted to each case; and to promote anticoagulation control.^6^^,^^7^

Clinical trials have shown that the new oral anticoagulants, also known as target-specific anticoagulants, have at least similar impact on the reduction of thromboembolic events, compared with warfarin, with better safety profiles.^8^^,^^9^^,^^10^^,^^11^^,^^12^ Important additional advantages include convenience, since there is no need to monitor the INR and thus no further consultations except for the routine medical follow-up; and fewer interactions: they present lack of susceptibility to dietary interactions and reduced susceptibility to drug interactions.^8^^,^^9^^,^^10^^,^^11^^,^^12^

There are few data comparing the cost of these drugs with actual costs of patients in clinical practice in Brazil.

## OBJECTIVE

The objective of this study was to perform an economic analysis comparing new oral anticoagulants versus warfarin, from the perspectives of Brazilian society and the public healthcare system, using real data from an anticoagulation clinic.

## METHODS

This was a cost-minimization analysis, using data from a cohort of patients of an anticoagulation clinic of Hospital Municipal Odilon Behrens, a public hospital in Belo Horizonte. This study was approved by the Research Ethics Committee of Hospital Municipal Odilon Behrens, and was conducted in accordance with the Helsinki Declaration. All patients provided written informed consent for their participation in the study.

### Patients and setting

All patients registered at this anticoagulation clinic between August and October 2011 were recruited for this study. The service provided by this clinic operates exclusively through the Brazilian public healthcare system (Sistema Único de Saúde, SUS), and its clientele consists mostly of patients of low socioeconomic status and low educational level.^13^

This anticoagulation clinic was established in 2001. Patients from the emergency department and hospital inpatient units with indications for oral anticoagulation are referred to this clinic for follow-up. On the day of the consultation, patients arrive at the hospital earlier, for blood collection for measurement of prothrombin time, which is expressed as the international normalized ratio (INR), using the calibration standardized by the World Health Organization (WHO) in 1982.^14^

While awaiting the test result and consultation, they participate in group educational activities, where they receive guidance on indications, risks and benefits of anticoagulation, and on interactions with food and diet. These educational activities are additional to the personalized educational activities that take place during the consultation. Although group educational activities are not formally recommended through guidelines for patient care in cases of anticoagulant use, this strategy is used in this anticoagulation clinic, because it has been shown to improve the time in therapeutic range (TTR).^15^

At the time of this study, the anticoagulation clinic was operating in four shifts per week, with the participation of one physician with overall responsibility, two or three residents, one pharmacist and one nurse. In addition, the clinic also had a secretary, who organized the records and service.

At each visit, the INR was assessed, the factors that interfere with anticoagulation control were identified and any dose adjustments needed were made, in accordance with a protocol based on guidelines for patient care in cases of anticoagulant use.^14^ Patient counseling was reinforced and the next visit was scheduled. The interval between consultations varied from less than one week to up to four weeks, depending on the INR result and whether there were any hemorrhagic complications, in accordance with the guidelines at that time.^14^ When the INR was within the therapeutic range, the next visit was scheduled for one week later and, successively, periods of one week were added to this interval, as long as the INR was still within the therapeutic range, up to four weeks. Thus, the consultation was exclusively for the purpose of anticoagulation control and there was no action towards underlying disease control or comorbidities, which were at the discretion of the attending physician (primary care physician, internal medicine physician, cardiologist, hematologist, etc.).

### Data collection and follow-up

Upon enrollment, patients were interviewed using a standardized questionnaire, and their records were reviewed, in order to obtain clinical, demographic and cost data: age, anticoagulation indication, risk factors and comorbidities, disability, occupation, salary, means of transportation to the anticoagulation clinic, home address, need to attend the clinic with a companion, companion’s occupation and salary. The categories of employment status used were: employee (defined as a person working for an employer, person or entity, receiving in return a cash compensation, including domestic workers),^16^ self-employed (defined as a person who was the owner of his business),^16^ unpaid worker or unemployed.

All patients were followed up for a minimum period of four weeks (maximum interval between the consultations, in accordance with the protocol), with assessment of INR tests, warfarin dosage, thromboembolic or hemorrhagic complications and hospitalizations. The data-gathering for this study did not affect the frequency of consultations or warfarin dosage, which were both at the attending physician’s discretion.

The quality of anticoagulation control was assessed by calculating the length of TTR, using the linear interpolation method of Rosendaal.^17^ The CHADS2-Vasc score, which is a clinical prediction rule for estimating the risk of stroke in patients with non-valvular atrial fibrillation, was used too.

### Assessment of costs

Cost assessment was performed by accounting for all the expenses involved in anticoagulation for the patients of the cohort. The costs were classified into two categories: direct and indirect (Table 1). Direct costs included operating costs relating to maintenance of the anticoagulation clinic: salaries of professionals working at the clinic (according to the hours devoted to this activity), cost of INR examination and cost of the drug (warfarin), according to the dose used. Indirect costs were those that were unrelated to the operation of the clinic, and included: patient transportation costs to the clinic, companion’s transportation expenses (in the case of patients who attended the consultation with a companion) and opportunity cost.

**Table 1.**
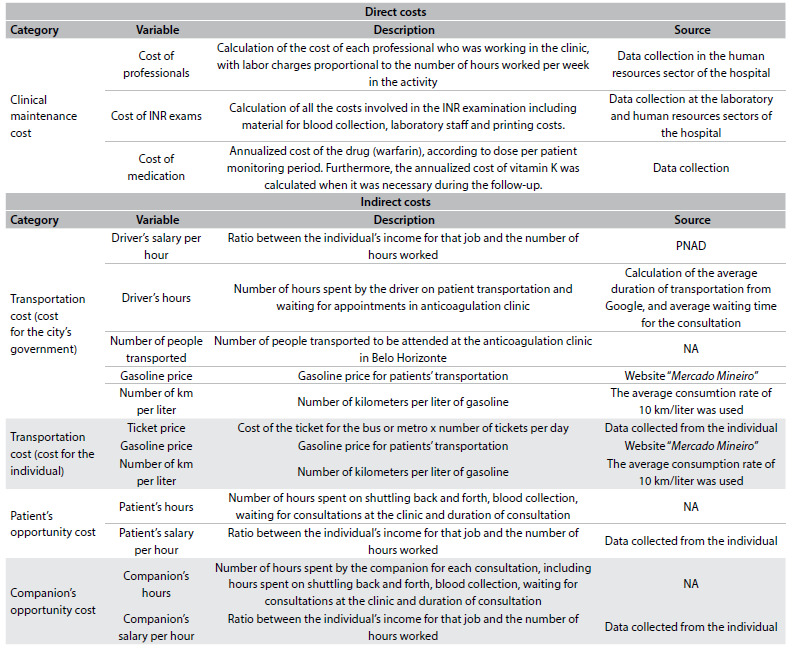
Description of the costs included in the cost-minimization analysis

PNAD = Pesquisa Nacional por Amostra de Domicílios; NA = not applicable.

In order to calculate the transportation cost, the means of transport used by each patient to attend the consultations was taken into account. For those who needed public transportation, we used the price of the bus ticket and the number of tickets used per day. For patients who used their own car, the distance from their home to the anticoagulation clinic was calculated (using Google Maps, available at www.google.com.br/maps) and, considering an average fuel consumption of 10 km per liter of gasoline and the average cost of gasoline (checked at the website www.mercadomineiro.com.br), the transportation cost was calculated. For patients who used the city’s patient transportation service, funded by the city government, the cost of fuel was taken into consideration, using the same calculation as above, and the cost of the driver’s salary. The cost of car rental was not calculated, since cities generally have their own cars for providing this type of service. Patients who needed taxi services were asked about their exact expenditure for their journey. To calculate the driver’s earnings per hour, data from the National Household Sampling Survey were used (Pesquisa Nacional de Amostra de Domicílios, PNAD).^16^^,^^18^ Through PNAD, we obtained the amount of the average hourly wage, which formed the reference value for the parameter. The range of salary was constructed by taking into consideration a range of one to four minimum wages as the monthly remuneration and assuming a working week of 40 hours.^18^

The opportunity cost refers to the amount of income from work that the individual failed to earn,^18^ or the cost to the individual of absence from work, through attending the consultation at the anticoagulation clinic.

The costs were calculated as the prices of July 2013, and were inflated in accordance with the Consumer Price Index (Índice de Preços ao Consumidor Amplo, IPCA) and converted to US dollars (USD) on August 19, 2015 (1 USD = R$ 3.486). In this study, all costs are expressed in US dollars.

### Cost-minimization analysis

Since apixaban, dabigatran and rivaroxaban have been shown to be not inferior to warfarin in randomized clinical trials among patients with atrial fibrillation and patients with venous thromboembolism,^6^^,^^7^^,^^9^^,^^19^^,^^20^ the present study used cost-minimization analysis. This method is a type of cost-effectiveness analysis that only compares two or more medical intervention costs, since the health outcomes resulting from the medical interventions compared are similar.^21^

In Brazil, apixaban, dabigatran and rivaroxaban are authorized by the National Health Surveillance Agency (Agência Nacional de Vigilância Sanitária, Anvisa) for use in patients with atrial fibrillation for prevention of cardioembolic events. Only dabigatran and rivaroxaban are authorized for use among patients with deep venous thrombosis and/or pulmonary embolism. To calculate the average cost of apixaban, dabigatran and rivaroxaban, we used the average price data for the period from January 1 to August 19, 2015, from the federal government’s drug purchasing website, which lists the prices of drugs for public institutions.^22^ Edoxaban was not included, because it has not yet been approved by Anvisa for use in Brazil.

## RESULTS

During the study period, 645 patients were registered in the anticoagulation clinic. Of these, 12 refused to participate. Thus, this study included 633 patients with a median age of 62 years (interquartile range 49-73), among whom 53.9% were elderly patients (≥ 60 years) and 59% were women. Table 2 illustrates the indications for anticoagulation among the patients included.

**Table 2.**
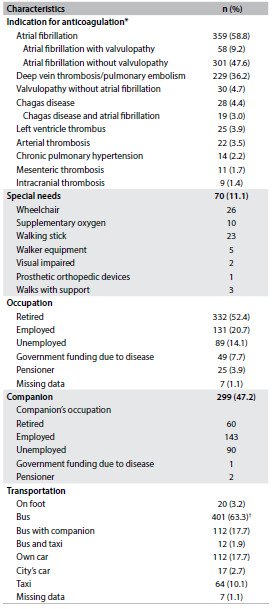
Characteristics of patients included (n = 633)

*Patients could have more than one indication for anticoagulation; ^†^Out of the 412 patients who used a bus, 63.6% used two buses, 29.6% used four buses and 4.4% used six buses per day to attend the consultation at the anticoagulation clinic.

Among the patients with non-valvular atrial fibrillation (n = 246), the CHADS2-Vase score was 1 for 1.2% of them, 2 for 8.9%, 3 for 20.3%, 4 for 27.2%, 5 for 22.0%, 6 for 15.4%, 7 for 3.3% and 8 for 0.8%. In two cases, we could not obtain all the information needed to calculate the CHADS2-Vasc score.

The mean follow-up period was 64 ± 28 days. The average proportion of the time in therapeutic range was 69.2% ± 25.0% (median 71.2%, interquartile range 52.8 to 92.4%), and 65.7% of the patients were within the therapeutic range for greater than or equal to 60% of the time. During the follow-up, 2.7% of the patients required administration of vitamin K and 1.6% had an episode of minor bleeding (hematoma, epistaxis, gingival bleeding or increased menstrual flow, without the need for hospitalization and/or transfusion of blood components). Two patients had gastrointestinal bleeding and one patient had hematuria. One patient (75 years old, male) presented an embolic event (ischemic stroke) during the follow-up, with INR at admission of 1.94.

Table 3 shows the total annualized costs for the 633 patients included. The average cost per patient per month was $ 54.26. Direct costs accounted for 32.5% of the total cost. Of these, 69.5% were costs relating to healthcare professionals, 21.8% to INR tests and 8.7% to warfarin. With regard to indirect costs (67.5% of the total), 52.4% were related to absenteeism from work and 47.6% to transportation to the clinic.

**Table 3.**
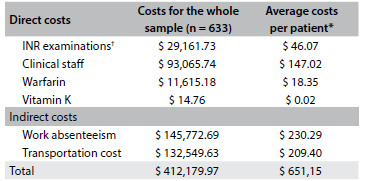
Annualized direct and indirect costs relating to patients at the anticoagulation clinic who were using warfarin (n = 633)

All costs are in US dollars. INR = international normalized ratio. *The costs over the same period for a patient using the new oral anticoagulants would be: $ 598.44 for apixaban, $ 616.80 for dabigatran and $ 625.92 for rivaroxaban; ^†^When the data from the study follow up is annualized, each patient would undergo 28 INR examinations on average.

**Figure 1.**
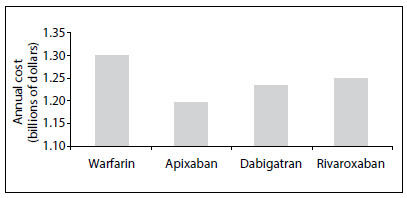
Graphical representation of average monthly cost of the strategy of using warfarin, at a public anticoagulation clinic, versus using apixaban, dabigatran and rivaroxaban, from data available on the federal government’s drug purchasing website, projected for the Brazilian population. Considering a population of 200 million inhabitants (according to the Instituto Brasileiro de Geografia e Estatística, at www.ibge.gov.br) and an estimated prevalence of atrial fibrillation of 1%, around 2 million patients need to make use of anticoagulants.

According to data from the federal government’s drug purchasing website, the average prices of apixaban, dabigatran and rivaroxaban for the public institutions from January 1st to August 19th, 2015,^20^ respectively, were $ 49.87, $ 51.40 and $ 52.16 per month, respectively (Table 4). Figure 1 provides a graphical representation of a projection of this difference for the Brazilian population.

**Table 4.**
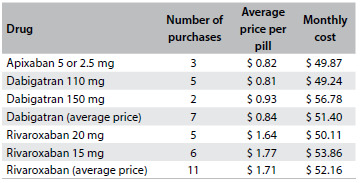
Average price for the new oral anticoagulants to public institutions

Source: federal government’s drug purchasing website, from purchases made between January 1 and August 19, 2015.^20^ Prices are in US dollars. Number of purchases = number of purchases of each dosage of each oral anticoagulant registered in the website, from the purchases made between January 1 and August 19,

### Sensitivity analysis

Table 5 illustrates the sensitivity analysis on the cost of anticoagulation using warfarin among elderly versus non-elderly patients, according to origin (Belo Horizonte versus other cities), distance from the home to the anticoagulation clinic (greater or less than 20 kilometers and greater or less than 30 kilometers) and indication (atrial fibrillation versus other indications).

**Table 5.**
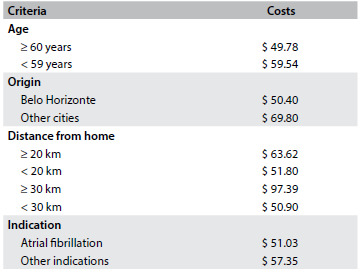
Sensitivity analysis on average monthly cost per patient using warfarin, at the anticoagulation clinic

Prices are in US dollars.

## DISCUSSION

Population ageing and increasing prevalence of chronic diseases, including hypertension and diabetes, which are factors that raise the risk of stroke among patients with atrial fibrillation, in addition to the higher prevalence of atrial fibrillation in the population,^24^ point towards growth in the numbers of patients with indications for anticoagulation. In clinical practice in Brazil, only a small proportion of patients receive anticoagulant therapy and this requires a solution.^5^ International guidelines recommend atrial fibrillation screening among patients greater than or equal to 65 years of age.^25^ However, within the Brazilian context, patients with recognized atrial fibrillation have not been treated with the recommended therapy for stroke prevention. Therefore, we witness patients being admitted to emergency departments presenting the disastrous consequences of strokes, which are generally more extensive and therefore associated with greater morbidity and mortality than those in patients without atrial fibrillation.^3^^,^^26^

The oral anticoagulant therapy currently available in the public healthcare system in Brazil is warfarin. It is recommended that the care for patients using oral anticoagulant therapy with warfarin should be provided in anticoagulation clinics,^14^ because these have been shown to be more effective in controlling these patients’ coagulation in terms of efficacy and safety. However, given the numbers of patients with indications for anticoagulation, creation of the required number of clinics to attend the Brazilian population is not feasible. Without these clinics, the decision to indicate anticoagulation and monitoring of patients is generally at the discretion of primary care physicians, who often do not have specific training for this and have limited access to continuing education.^27^^,^^28^ Thus, they feel insecure with regard to making such decisions and do not have enough time to provide the number of consultations that anticoagulation control requires.^14^ Additionally, control undertaken at primary care centers may produce results that are inferior to those at anticoagulation clinics,^23^ especially considering that performing the blood analysis work in different laboratories with interference from preanalytical factors and/or without adequate standardization of thromboplastin, may lead to problems in measuring INR levels.^14^ Consequently, this can generate the need for more consultations and might increase the risk of complications, thereby further increasing the cost.

The present study shows that in the context of Brazilian healthcare, after calculating all the costs involved in controlling anticoagulation at an anticoagulation clinic among patients using warfarin, this strategy has a higher cost than the sale price of the new oral anticoagulants to the public institutions. These results are extremely important, considering the urgent need to act more effectively in primary and secondary prevention of cardioembolic stroke among patients with atrial fibrillation within the Brazilian context.^5^

The costs for consultations relating to the new oral anticoagulants were not calculated, since these drugs do not require further consultations, other than the usual controls among these patients. For both warfarin and the new oral anticoagulants, the patient needs to undergo clinical follow-up with the referral physician, since the anticoagulation clinic only assesses the oral anticoagulant therapy.

It is important to note that the strategy of using the new oral anticoagulants is not appropriate for all patients with indications for oral anticoagulation, since there is lack of evidence regarding the impact of the new oral anticoagulants for some conditions, for example, among patients with rheumatic valve disease and atrial fibrillation, prosthetic heart valves, Chagas cardiomyopathy and thrombus in the left ventricle. Some patient profiles have been excluded from clinical trials, such as cases of advanced kidney failure or liver failure; or have been underrepresented, such as cases of extremes of weight.

The sensitivity analysis showed that for some patient profiles, the strategy of using the new oral anticoagulants seems to be even more economically attractive. For example, among non-elderly patients, there is a higher cost relating to absenteeism from work than among elderly patients; and among patients living in cities other than where the clinic is located, or living at least 20 kilometers from the clinic, for whom the cost relating to transportation becomes more significant, thus making anticoagulation with warfarin a more expensive strategy. When comparing patients using anticoagulants because of atrial fibrillation with patients with other indications, the costs of taking warfarin were significantly lower among patients with atrial fibrillation ($ 51.03 versus $ 57.35). Thus, for patients with atrial fibrillation, the costs of taking warfarin are comparable to the costs of taking the new oral anticoagulants.

This study has some limitations. Cost-minimization analysis assumes equivalence between interventions. Clinical trials have shown that the new oral anticoagulants are at least as effective as warfarin, with a better safety profile. Thus, there is potential for even greater cost reduction with the new oral anticoagulants. Although this was a single-center study, the protocol used in this anticoagulation clinic is based on international guidelines for patient care with anticoagulant use. Additionally, in these analyses, we assumed that control undertaken at anticoagulation clinics is the recommended strategy for patients taking warfarin.^14^ However, control at anticoagulation clinics is not universally available for patients using warfarin, and this comparison may not apply to all settings.

## CONCLUSION

This cost-minimization analysis using real data from clinical practice found that in the Brazilian context, from the perspectives of the public healthcare system and of society, the calculated costs relating to warfarin use in anticoagulation clinics seem to be currently higher than those relating to the strategy of using the new oral anticoagulants. These data provide support for the discussion about incorporating these new drugs for patients within the public healthcare system, with the potential to reduce the incidence of systemic embolic events and to bring even greater savings from a financial point of view and in terms of public health.
